# MRI-negative Cushing’s Disease: Management Strategy and Outcomes in 15 Cases Utilizing a Pure Endoscopic Endonasal Approach

**DOI:** 10.1186/s12902-022-01069-5

**Published:** 2022-06-09

**Authors:** Guive Sharifi, Amir Arsalan Amin, Mohammadmahdi Sabahi, Nikolas B. Echeverry, Nader Akbari Dilmaghani, Seyed Ali Mousavinejad, Majid Valizadeh, Zahra Davoudi, Badih Adada, Hamid Borghei-Razavi

**Affiliations:** 1 Department of Neurosurgery, Loghman Hakim Hospital, Shahid Beheshti University of Medical Sciences, Tehran, Iran; 2 Skull Base Research Center, Loghman Hakim Hospital, Shahid Beheshti University of Medical Sciences, Tehran, Iran; 3 Neurosurgery Research Group (NRG), Student Research Committee, Hamadan University of Medical Sciences, Hamadan, Iran; 4 Department of Neurosurgery, Rutgers-New Jersey Medical School, Newark, NJ USA; 5 Department of Otolaryngology, Head and Neck Surgery, Loghman Hakim Hospital, Shahid Beheshti University of Medical Sciences, Tehran, Iran; 6 Obesity Research Center, Research Institute for Endocrine Sciences, Shahid Beheshti University of Medical Sciences, Tehran, Iran; 7 Department of Endocrinology, Loghman Hakim Hospital, Shahid Beheshti Medical University, Tehran, Iran; 8 Department of Neurological Surgery, Pauline Braathen Neurological Center, Cleveland Clinic Florida, Weston, Florida, USA; 9 Cleveland Clinic Lerner College of Medicine of Case Western Reserve University, Director of Minimally Invasive Cranial and Pituitary Surgery Program, Research Director of Neuroscience Institute, Cleveland Clinic Florida Region, 2950 Cleveland Clinic Blvd. Weston, Cleveland, FL 33331 USA

**Keywords:** Negative MRI, Cushing’s disease, Endoscopic transsphenoidal surgery, Outcome, Management

## Abstract

**Background:**

Cushing’s disease (CD) is among the most common etiologies of hypercortisolism. Magnetic resonance imaging (MRI) is often utilized in the diagnosis of CD, however, up to 64% of adrenocorticotropic hormone (ACTH)-producing pituitary microadenomas are undetectable on MRI. We report 15 cases of MRI negative CD who underwent surgical resection utilizing a purely endoscopic endonasal approach.

**Methods:**

Endoscopic endonasal transsphenoidal surgery (EETS) was performed on 134 CD cases by a single surgeon. Fifteen cases met inclusion criteria: no conclusive MRI studies and no previous surgical treatment. Data collected included signs/symptoms, pre- and post-operative hormone levels, and complications resulting from surgical or medical management. Data regarding tumor diameter, location, and tumor residue/recurrence was obtained from both pre- and post-operative MRI. Immunohistochemistry was performed to assess for tumor hormone secretion.

**Results:**

Aside from a statistically significant difference (*P* = 0.001) in histopathological results between patients with negative and positive MRI, there were no statistically significant difference between these two groups in any other demographic or clinical data point. Inferior petrosal sinus sampling (IPSS) with desmopressin (DDAVP®) administration was performed on the 15 patients with inconclusive MRIs to identify the origin of ACTH hypersecretion via a central/peripheral (C/P) ratio. IPSS in seven, five and three patients showed right, left, and central side lateralization, respectively. With a mean follow-up of 5.5 years, among MRI-negative patients, 14 (93%) and 12 patients (80%) achieved early and long-term remission, respectively. In the MRI-positive cohort, over a mean follow-up of 4.8 years, 113 patients (94.9%) and 102 patients (85.7%) achieved initial and long-term remission, respectively.

**Conclusions:**

Surgical management of MRI-negative/inconclusive Cushing’s disease is challenging scenario requiring a multidisciplinary approach. An experienced neurosurgeon, in collaboration with a dedicated endocrinologist, should identify the most likely location of the adenoma utilizing IPSS findings, followed by careful surgical exploration of the pituitary to identify the adenoma.

## Introduction

Cushing’s disease (CD) is the most common cause of hypercortisolism [1]. Left untreated, CD can result in multiple complications, most often cardiovascular disease or infection, and has a mortality rate 1.7–4.8-times higher than the general population [2–4]. Although MRI is the imaging modality of choice for identifying these tumors, imaging is often inconclusive [5].

Prior studies have shown that adrenocorticotropic hormone (ACTH)-producing pituitary microadenomas are undetectable on MRI in 36–64% of cases [5]. However, the development and widespread utilization of 3-T MRI (3TMRI) has led to much higher tumor detection rates [6, 7]. With a negative predictive value of approximately 19–94% and variable sensitivity and specificity, anywhere from 4 to 54% of MRIs are incorrectly reported, especially in the setting of ACTH-secreting pituitary adenomas [8, 9]. With such variation in radiographic appearance, reliance on imaging for the management of CD patients can cause significant uncertainty for neurosurgeons and endocrinologists alike.

The choice approach in the surgical management of these adenomas is via an endoscopic endonasal transsphenoidal surgery (EETS) [2, 10, 11], resulting in overall post-operative remission rates of 64–93% globally and 50–71% for cases without a conclusive MRI [12–15]. Inconclusive MRIs pose a significant challenge in the surgical management of CD, with the decision to pursue surgery for MRI-negative CD remaining highly controversial [8, 10, 14, 16]. In this study, we report 15 cases of CD without positive MRIs who underwent adenoma resection via EETS.

## Patients, materials and methods

### Patients population

Between January 2005 and December 2018, EETS was performed in 134 CD cases by a single surgeon at Loghman hakim and Erfan hospitals. Of those patients, 15 cases met inclusion criteria: inconclusive MRI studies and no prior surgical treatment. The population consisted of 12 women (mean age 32.5 years; range 14–65 years) and 3 men (mean age 35 years; range 22–60 years). Data collected included signs/symptoms, pre- and post-operative hormone levels, and complications resulting from surgical or medical management. Data regarding tumor diameter, location, and tumor residue/recurrence was obtained from both pre- and post-operative MRIs. Immunohistochemistry was performed to assess for tumor hormone secretion.

### Ethics approval and consent to participate

All procedures performed in this study involving human participants were in accordance with the ethical standards and approved by the Shahid Beheshti Medical University (SBMU) Ethical Committee and the 1964 Helsinki Declaration and its later amendments or comparable ethical standards. Also, a written informed consent was obtained from all subjects (or their parent or legal guardian in the case of children under 16).

### Imaging

All patients underwent pre- and post-operative dynamic pituitary MRI via a superconducting 1.5-T scanner. Prior to gadolinium injection, T1-weighted Spin Echo (SE) and T2-weighted turbo SE images, followed by coronal dynamic acquisition (T1-weighted turbo SE), were obtained in the coronal plane using the following protocol: TR/TE, 400/20 ms; 288 · 192 matrix; two excitations; 18 · 18 cm field of view (FOV); 3 mm in thickness with 0.3-mm intersection gap. Afterwards, with simultaneous gadolinium injection, coronal and sagittal T1-weighted SE images were obtained 2 minutes following injection. All images were independently reviewed by both a radiologist and a neurosurgeon.

MRIs studies were categorized into direct and indirect signs of CD. Direct signs consisted of any inhomogeneity found in the pituitary, such as a lesions with diminished enhancement. Indirect signs included pituitary stalk deviation and bulging or erosion of the sellar contour. MRI studies were considered negative (normal) if no direct or indirect signs were identified.

In some cases, small lesions with diameters under 6 mm may be seen on MRI however are not considered indicative of CD due to the high prevalence of incidentalomas in this region. MRIs in which these lesions were present were classified as inconclusive.

Any uncertainty in interpreting the MRIs by any of the reviewers resulted in exclusion of the image from this study.

### Pre-operative endocrine examination

All cases were ACTH-dependent Cushing syndrome showing clinical features including weight gain, proximal myopathy, and wide base purple striae. Furthermore, all cases demonstrated laboratory abnormalities consistent with CD, including increased 24-hour urinary free cortisol (UFC) excretion, loss of the cortisol circadian rhythm, high basal ACTH level, failure of low-dose dexamethasone to suppress cortisol secretion in addition to serum suppression or 24-hour UFC after high-dose dexamethasone. Additionally, pre- and post-operative levels of anterior pituitary hormone including prolactin, growth hormone (GH), insulin-like growth factor I (IGF-I), thyroid stimulating hormone (TSH), free/total Triiodothyronine (T3)/ Thyroxine (T4), follicle-stimulating hormone (FSH), Luteinizing hormone (LH), and free/total testosterone (men) or estradiol (premenopausal women) were measured.

The 15 cases of MRI negative CD were diagnosed and categorized according to their endocrine profile in order to distinguish the ACTH-dependent CD from pseudo-cushing syndrome.

### Bilateral inferior petrosal sinus sampling (BIPSS)

All 15 cases of MRI-negative ACTH-dependent Cushing’s syndrome underwent bilateral inferior petrosal sinus sampling (BIPSS). To confirm that the elevated ACTH secretion originated from the pituitary, BIPSS was simultaneously performed with central/peripheral (C/P) ACTH gradient measurement, utilizing the calculations described by Oldfield et al. [17].

No significant complications occurred in performing the procedures. A petrosal to peripheral ACTH ratio ≥ 2.0 in the basal state, a peak ratio ≥ 3.0 after desmopressin (DDAVP®) administration, or a normalized IPS:P ratio > 0.8 were considered diagnostic of CD. Additionally, tumor lateralization was specified when the interpetrosal gradient ratio of ACTH was ≥1.4 [18].

### Endoscopic Endonasal Transsphenoidal surgical approach

**Fig. 1:**
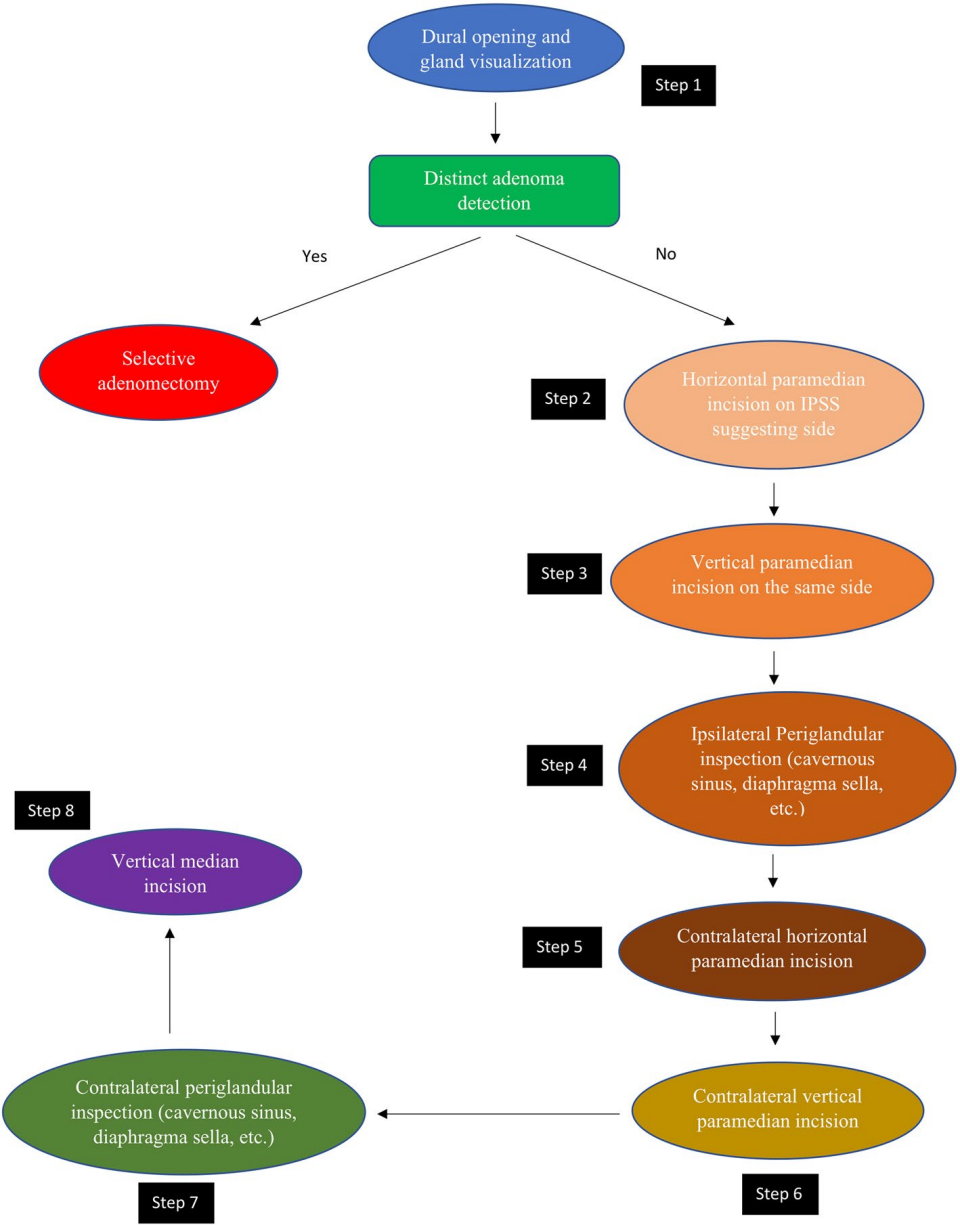
Eight-step MRI negative Cushing’s disease surgical management

**Fig. 2:**
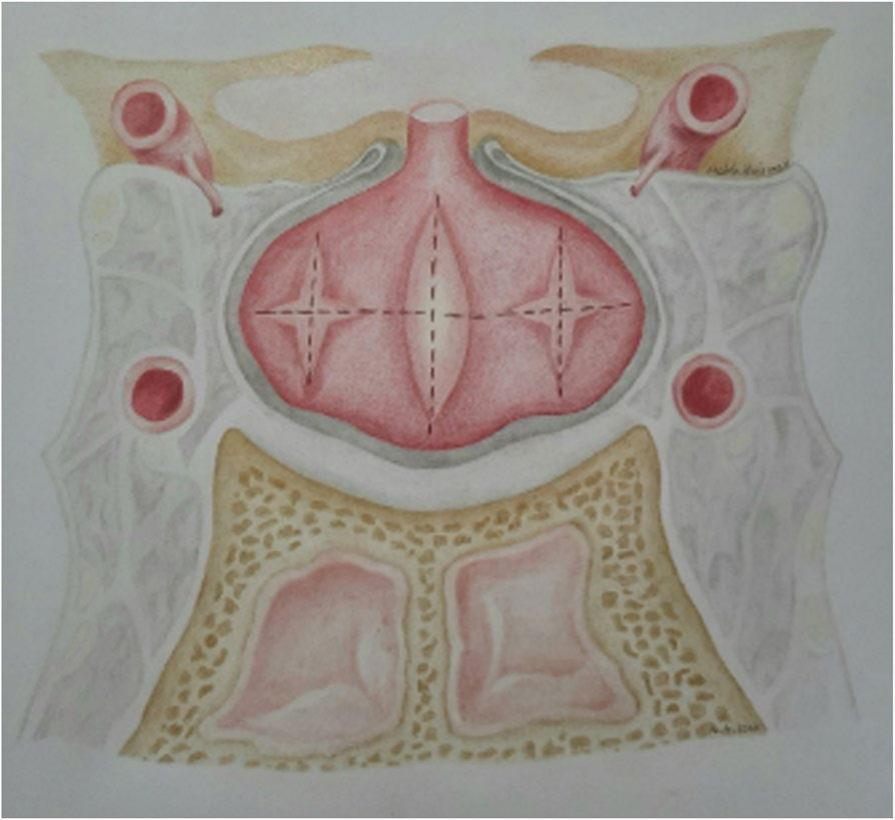
Schematic illustration of 8 steps in endoscopic endonasal approach to MRI inconclusive Cushing’s disease (Resembling half Georgia flag)

All patients underwent surgery by a single neurosurgeon and otolaryngologist (ENT) with extensive experience in pituitary tumor excision via EETS. Exposure to the sellar floor was provided by an ENT surgeon while drilling of the sella was performed by the neurosurgeon. Extensive drilling of the sellar floor laterally up to the carotid artery bilaterally provided a wide view of the medial wall of the cavernous sinus as well as exposure of the anterior and posterior intercavernous sinuses was performed in all cases. The dura was then opened to expose the pituitary gland. Following tumor identification, adenomectomy was performed with selective removal of a rim of normal pituitary tissue. In cases where a tumor was not visualized on initial exposure of the pituitary, the pituitary gland was explored laterally via a horizontal paramedian incision on the IPSS suggesting side. If a tumor was not visualized at this stage, a vertical paramedian incision was then performed. In some cases, a cream-like substance was drained from the pituitary incision. Although this was suspicious of a tumor and tissue biopsy was obtained, it was not considered a definite tumor diagnosis and thus surgical exploration (EXP) was done in the same manner on the other side of the pituitary. In the scenario where no distinct adenoma was found, both sides of the pituitary gland underwent EXP with emphasis on lateralizing sides distinguished by IPSS. However, we did not rely solely on IPSS lateralization, as whole gland EXP was performed in all cases. Although ACTH secreting pituitary adenomas are the most common cause of Cushing syndrome, pituitary adenomas can also be ectopic, forming outside of the sella turcica with no direct connection to the pituitary gland [19]. After EXP of each side of the gland, ipsilateral periglandular inspection with visualization of the medial wall of the cavernous sinus and diaphragm was performed to identify a potential ectopic microadenoma in the periglandular region. Although the exact origin of ectopic ACTH-producing pituitary adenomas is unclear, they likely emerge from remnants of Rathke’s pouch during its development course [20]. As a result, these tumors can be discovered in the nasopharynx, sphenoid sinus, cavernous sinus, clivus, or suprasellar area [21]. Detecting an adenoma at this stage may prevent further unnecessary EXP of pituitary gland. If a visible tumor was still not detected, a vertical medial incision was made on the pituitary gland adjacent to the pituitary stalk and neurohypophysis. If a tumor could not be reliably identified by extensive EXP of the entire pituitary gland or BIPSS failed to localize a pituitary adenoma, we did not progress to performing incomplete or complete hypophysectomy. Figures 1 and 2, respectively, demonstrate the surgical management algorithm and pituitary incisions for MRI-negative CD.

If an ectopic ACTH-secreting adenoma is not easily found, permanent destructive or ablative surgeries such as bilateral adrenalectomy and hypophysectomy may be required [20]. Despite the danger of Nelson syndrome, bilateral adrenalectomy remains a feasible option in the management of refractory CD [22, 23].

### Histological examination

All intraoperative tissue specimens obtained underwent histological examination by a pathologist. Pituitary specimens were fixed in buffered 10% formalin and embedded in paraffin wax. All specimens were first examined by Hematoxylin and Eosin (H&E) staining to detect regions which had loss of acinar organization. Additionally, reticulin and periodic Acid-Schiff (PAS) staining was implemented for a more accurate histopathologic diagnosis. Immunohistochemistry staining was used to identify cytokeratin and anterior pituitary hormones, including ACTH, in the case of a pituitary adenoma not being detected by H&E staining. The presence of ACTH-secreting cells was examined via immunocytochemistry using specific anti-ACTH antibodies.

### Post-operative endocrinologic assessment and follow up

Serum cortisol and ACTH levels were monitored for 2–5 days following surgery. Initial follow-up occurred 2 weeks post-operatively with a subsequent visit occurring 3 months postoperatively, during each visit a complete pituitary hormonal evaluation was performed. This evaluation was repeated every 3 months for up to 2 years and every 6 months after that. An initial postoperative pituitary MRI was typically performed within 3 months after surgery. For patients to be considered to be in initial post-operation remission, a basal plasma cortisol level lower than 140 nmol/L (5 μg/dL) or adequate suppression of plasma cortisol (≤56 nmol/L) (≤1.8 μg/dL) following the 1-mg dexamethasone suppression test was necessary during the first month following surgery. Long term remission was defined as a plasma cortisol lower than 84 nmol/L (3 μg/dL) after a 1-mg dexamethasone suppression test at the final visit. Recurrence was defined as a recurring case of hypercortisolism with insufficient suppression of plasma cortisol (> 140 nmol/L) after a 1-mg dexamethasone suppression test. Clinical criteria for remission included significant symptomatic improvement or resolution without additional therapy (radiotherapy, adrenalectomy). Patients achieving remission had to meet both laboratory and clinical criteria to be classified as such. Glucocorticoids were not given postoperatively except when there was laboratory evidence of hypercortisolism and/or clinical manifestations of glucocorticoid insufficiency. Additionally, 4 to 6 weeks post-operatively, thyroid and gonadal axis function was assessed by measuring free T4, TSH, FSH, and LH levels in addition to end-organ hormones (estradiol in women and testosterone in men).

### Statistical analysis

SPSS software (version 26, Chicago, IL) was used to analyze the data. For continuous data, we calculated descriptive statistics, mean and standard deviation (SD), and for categorical variables, frequency and percentages were calculated. The chi-square or Fisher’s exact test was used to analyze categorical data, while the student’s t-test or Mann- Whitney U test was used to analyze continuous variables’ means, depending on the distribution’s normality. Statistical significance was defined by a *p* value of < 0.05.

## Results

Demographic and clinical data of 134 patients with CD who underwent EETS are shown in Table 1. Fifteen (11.2%) of the 134 CD patients who underwent EETS were MRI-negative and 119 patients (88.8%) were MRI positive. The female/male ratio in the MRI-negative group was four to one while this ratio in the MRI-positive cohort was 2.6. With regards to sex distribution, Fisher’s exact test found no statistically significant difference between these two groups (*P =* 0.565). All patients had clinical manifestation of Cushing’s syndrome including obesity, hirsutism, glucose intolerance, and hypertension. As shown in Table 1, pre-operative ACTH level was 134.02 ± 21.78 ng/l and 151.76 ± 44.17 ng/l in MRI-negative and MRI-positive patients, respectively, and no statistically significant difference was observed between these two groups (*P =* 0.781). As demonstrated in Table 1, UFC was 462.3 ± 43.98 μg/24 h and 478.4 ± 73.02 in MRI-negative and MRI-positive patients, respectively, and no statistically significant difference was observed between these two groups (*P =* 0.832).

**Table 1:** Demographic and clinical data

	MRI-negative	MRI-positive
Number of patients	15	119
Gender (F/M)	12/3	86/33
Age (years)	F: 36.16 ± 12.34 (14–65)	F: 34.24 ± 15.46 (15–72)
	M: 20.66 ± 8.25 (14–25)	M: 24.32 ± 10.29 (16–59)
Mean UFC (μg/24 h)	462.3 ± 43.98	478.4 ± 73.02
ACTH (ng/l)	134.02 ± 21.78	151.76 ± 44.17
Follow up period: range (month)	61.3 ± 11.3	58.3 ± 10.7

*Abbreviations. F* Female, *M* Male, *UFC* Urinary free cortisol, *ACTH* Adrenocorticotropic hormone

IPSS with DDAVP® administration was performed on the 15 MRI-negative patients to identify the origin of ACTH hypersecretion via the C/P ratio. Seven patients showed right-sided lateralization and five patients showed left-sided lateralization. In remaining three patients, IPSS did not show an ACTH interpetrosal gradient ratio greater than the cutoff point, which was interpreted as an ACTH hypersecretion with central origin. On EXP, adenomas were found in 2 of the 3 patients, with no adenoma being found in the 3rd. The IPSS results were in concordance with our observations during EXPs in 60% of patients. However, in 13% of patients, no adenoma was detected, and in 26% an adenoma was found on the opposite side of the pituitary where pre-operative IPSS results initially reported a tumor or was suggestive of one being present. In 60% of MRI-negative patients, histological examination demonstrated an adrenocorticotropic pituitary adenoma, but in 40% no adenoma was found after pathological examinations. In MRI-positive patients, positive histology was observed in 112 patients (94.1%), while in 7 patients (5.9%) histopathological studies were negative. Fisher’s exact test revealed that the difference between MRI-negative and MRI-positive patients in terms of histopathological result was statistically significant (*P =* 0.001).

In all four patients who had discordant IPSS results as well as the patients who had negative or inconclusive findings on EXP, tissue samples were obtained from suspicious sites during EXP and were sent for histopathological examination. Histopathology demonstrated adrenocorticotropic adenoma tissues in 3 of them on the opposite side of the IPSS suggested region, while in 1 of them the histological results were inconclusive. This patient (case 10) achieved initial remission, however she experienced recurrence after 25 months, and similarly to her initial presentation, MRI findings were negative and IPSS suggested right sided lateralization. She underwent revision surgery, and a distinct adenoma was detected on the right side, which was confirmed by histological examination, after which she went into remission following selective adenectomy (Table 2).

**Table 2:** Presents summary of patients’ demographics, IPSS and surgical exploration results

patients	sex	age	IPSS	exploration	IPSS/exp	Positive adenoma cell on histology	recurrence
1	F	36	R	L	Discordant	Yes (on left side sample)	
2	F	33	L	No adenoma		Yes	
3	M	14	R	R	concordant	Yes	
4	F	60	C	C	concordant	No	
5	F	50	L	L	concordant	Yes	
6	F	32	C	No adenoma		Yes	
7	F	23	L	L	concordant	Yes	
8	M	25	R	R	concordant	No	
9	F	22	C	C	concordant	No	
10	F	35	R	L	Discordant	No	Recurrence after 25 months
11	F	26	L	R	Discordant	Yes (on right side sample)	Recurrence after 19 months
12	F	65	R	R	concordant	No	
13	F	38	R	R	concordant	Yes	No remission
14	M	23	L	R	Discordant	Yes (on right sample)	
15	F	14	R	R	concordant	No	

*Abbreviations: F* Female, *M* Male, *IPSS* Inferior petrosal sinus sampling, *EXP* Surgical exploration, *R* Right, *L* Left

Among the patients with inconclusive MRI, 14 (93%) achieved initial remission, 12 of which (80%) went on to long term remission with a mean follow up of 5.5 years. Two patients (cases 10 and 11) developed recurrence following initial remission; according to the IPSS suggested side, partial hypophysectomy was performed in both cases however neither was able to achieve remission afterwards. One patient (case 13) was unable to achieve initial remission following the initial surgery and thus required continued medical management. With a mean follow-up of 4.8 years among the 119 patients with positive MRI, 113 patients (94.9%) and 102 patients (85.7%) achieved initial and long-term remission, respectively. There were no statistically significant differences between these two groups in terms of either initial (*P =* 0.767) or long-term remission (*P =* 0.457). Among the 102 patients who achieved long-term remission, 12 patients (11.7%) experienced disease recurrence. With regards to recurrence rate, there was no statistically significant difference between patients with either positive or negative MRI (*P =* 0.542).

In two patients (cases 2 and 6) the adenoma was not found during EXP, however tissue samples obtained from the IPSS suggested side demonstrated adrenocorticotropic pituitary adenoma in both patients on histopathological examination.

Diabetes insipidus (DI) was the most frequent complication associated with CD. Transient DI occurred in seven cases with resolution prior to discharge. There was one case of permanent DI diagnosed in follow-up. Additionally, one patient developed symptomatic adrenal insufficiency requiring glucocorticoid replacement. Two patients developed hypothyroidism requiring hormone replacement. Panhypopituitarism was not seen following the initial surgeries however occurred in one case following revision surgery (partial hypophysectomy) which required hormone replacement therapy. Cerebrospinal fluid (CSF) leak resulting in meningitis was seen in one patient, however no other complications occurred during the post-operative period. None of our patients demonstrated clinical or endocrinological signs of gonadal insufficiency in follow-up aside from the aforementioned case of panhypopituitarism following revision partial hypophysectomy. In the MRI-positive cohort, 51 patients showed transients DI (42.8%), with 4 of the patients (3.4%) experiencing DI till last follow-up. Partial anterior pituitary insufficiency and complete anterior pituitary insufficiency was observed in one (0.8%) and two (1.6%) patients, respectively. Syndrome of inappropriate antidiuretic hormone (SIADH) secretion was observed in 3 patients (2.5%).

## Discussion

In this study we present the outcomes of pure endoscopic endonasal surgical treatment of fifteen patients with MRI-negative Cushing’s disease. Due to the arduous nature of treatment in this patient population, we used a precise method of EXP as described above, resulting in initial remission in 93% of patients post-operatively. Based on the work of Bansal et al., patients with a definite adenoma on MRI who underwent microscopic transsphenoidal surgery had a statistically significant greater rate of early remission and lower rates of persistent disease than those with negative/equivocal findings [24]. However, in terms of late remission and recurrence, there was no statistically significant difference between these two groups [24]. Negative/equivocal MRI results and the incidence of macroadenoma, particularly in patients with cavernous sinus invasion, were found to predict poor remission rates [24]. According to some investigations, MRI-negative CD patients had a poorer remission rate [25, 26]. In other studies, however, there was no statistically significant difference in remission between those who had MRI-negative CD and those who had a MRI-positive CD, which is consistent with our result [14, 27–32]. Recurrence occurred in 2 patients, while 12 patients showed no clinical or endocrinological signs of recurrence during the mean follow-up of 5 years, and one patient did not go to remission. Aside from one CSF leak leading to meningitis and one case of permanent DI, there were no major surgery related complications. Pituitary CD is a common and potentially lethal condition that, if left untreated, can lead to sequelae such as morbid obesity, hypertension, and diabetes mellitus. Diagnosis and treatment of CD is more challenging than other functional pituitary adenomas. Currently, trans-sphenoidal pituitary EXP is considered the standard of care for CD [33–35]. CD is typically diagnosed by endocrinologist through clinical symptoms, and supported by laboratory tests such as the 8 AM blood or saliva cortisol level, 24 hours urinary free cortisol level, low- and high-dose dexamethasone suppression tests, and the corticotropin-releasing hormone (CRH) stimulating test [36–38]. When ACTH-dependent CD is diagnosed, or clinical signs and symptoms are highly suggestive of it, MRI imaging of the pituitary is often the next step to identify the causative agent i.e., a pituitary adenoma. With regards to pituitary lesions, MRI is considered the most sensitive imaging modality, however reported sensitivity varies significantly between studies, with reported rates ranging from 22 to 92% [39–41].

The rate of MRI-negative microadenomas is reported to be between 36 to 63% [5]. Hofmann et al. reported no identified tumor in 49.3% of 270 MRIs [29]. Yamada et al. reported a lower frequency (17%) of MRI-negative CD in their series [42]. In our series, only 15 out of 134 (11.19%) CD patients were MRI-negative. In general, negative-MRIs could be explained by several factors such as field strength, technique (the correct pulse sequence and parameters), radiologist interpretation errors, or tumor size. Identifying tumors smaller than 3 mm in diameter is difficult in MRIs with 2.5- to 3-mm-thick image sections [29]. Dynamic MRI and 3-TMRI can result in a higher sensitivity in identifying ACTH-secreting microadenomas [6, 7, 43]. In addition, spoiled gradient-recalled echo sequence (SPGR) view can help to increase sensitivity [44]. The relatively low number of negative-MRIs in our study can be attributed to the more extensive review of MRI images, utilization of high-field strength MRI (1.5 T), as well as the implementation of SPGR dynamic studies with 1.5- to 2.0-mm-thick sections, in addition to standard methods. Additionally, assessment of images by experienced pituitary neuroradiologists may have reduced the negative-MRI rate in our series. Although small tumor size is a likely factor in MRI-negative CD, prior studies have reported examples of MRI-negative microadenomas 4-6 mm in size, typically large enough to be easily identified on EXP [42].

If MRI is unable to identify the tumor definitively, the next best step is venous sampling to confirm CD. There are various indication for BIPSS, including patients who have clinical and laboratory findings of CD but normal or inconclusive MRI results [45], cases that do not have a clear hormone test response, or cases where there are inconsistencies between laboratory and imaging results [46]. BIPSS is also recommended by some as standard for any case of confirmed ACTH-dependent Cushing’s syndrome [47, 48]. In our institution, BIPSS is reserved for MRI-negative Cushing’s patients. Newell-Price et al. reviewed 21 studies with 569 total patients, and found that BIPSS with CRH stimulation had a 96% sensitivity and 100% specificity in separating CD from pseudo-Cushing’s states [49]. Most studies report a 90–100% sensitivity and specificity for BIPSS [50–52]. In the majority of cases of CD, a pituitary microadenoma can be found eccentric to one side of the pituitary, having venous drainage directly into the ipsilateral inferior petrosal sinus (IPS) [53].

This phenomenon is the basis for utilizing BIPSS as a means of lateralizing ACTH secreting pituitary tumors. There are many instances where EXP fails to detect a pituitary adenoma, despite conformation of pituitary origin of ACTH secretion via BIPSS. Evidence of lateralization prior to surgery can convince the surgeon to perform a guided hemi hypophysectomy. In our series, the accuracy of BIPSS for lateralizing adenomas was 60%, similar to the reported accuracy in the literature of approximately 70% [17]. Inaccurate lateralization from BIPSS has been attributed to asymmetrical venous drainage with shunting of blood toward the dominant side. Thus, BIPSS appears to be a superior diagnostic tool compared to other means of lateralization, and neurosurgeons should be wary of making operative decisions solely from BIPSS data [49].

The standard of care for MRI-negative CD is highly disputed. There is evidence suggesting surgical exploration is more problematic than watchful waiting [8], or that it is not indicated in MRI-negative CD [54]. Many advancements have led to the widespread adoption of transsphenoidal approach during the last three decades, especially the endoscope [31]. Regardless of the width or depth of access, the endoscopic approach allows the surgeon to have a large panoramic view. Many cases in the literature have reported successfully treating functional pituitary tumors via endoscopic surgery [27, 31, 55–58]. The results suggest that they are on par with, if not superior to, traditional microscopic approaches. When patients were operated on utilizing a microscopic technique assisted by a pre-operative ACTH gradient, the overall rate of partial adenomectomy (partial hypophysectomy) was 30%, including 19% in patients with positive MRIs and 40% in those with negative MRIs [28]. However, endoscopic visualization of pituitary adenomas has allowed for the need for partial adenomectomy to be reduced to less than 2%, limiting the damage to the normal pituitary gland during operation [28]. A recently published meta-analysis demonstrated that although there was no statistically significant differences between EETS and microscopic endonasal transsphenoidal surgery in the sub-analysis with regards to recurrence rate, remission rate, and persistence rate, the recurrence rate in the microscopic endonasal transsphenoidal surgery group was almost three times higher than in the EETS group [11]. As a result, EETS appears to be a possible suggested therapeutic method, while more studies are needed to establish the therapeutic method of choice [59].

In general, pituitary surgery is not advisable in cases of MRI-negative CD where IPSS is not able to prove a central origin of ACTH secretion [42]. However, when IPSS demonstrates central ACTH secretion, surgical intervention has been proposed as a first line treatment in MRI-negative CD [25, 32, 42, 60]. The outcome of surgical intervention in MRI-negative patients is variable in the literature. Some reports indicated lower remission rate in these patients [42, 61], while others have concluded that EXP results in greater complications in this population [8, 15]. Additionally, several studies have shown no significant difference in outcomes of pituitary surgery between MRI-negative and MRI-positive patients [14, 25, 32]. Pivonello et al. found the lack of tumor detection on pre-operative MRI operation to be a negative prognostic factor in surgical management [62]. In the present study, surgery was performed for all MRI-negative Cushing’s patients with positive IPSS results. We achieved 93% initial remission and 80% long term remission rates, comparable to mean remission rates in patients with preoperative identification of tumor, as reported in the literature, ranging from 52.6–100% [62].

Failure to identify an adenoma on EXP or in histologic examination is not uncommon in the surgical management of CD. Intraoperative detection of the adenoma has been shown to be a factor of favorable prognosis [63–65]. Similarly, failure to identify an adenoma on histopathology has been found to be a negative prognostic indicator. Specifically, remission rates were significantly lower in cases where no histological tumor identification could be provided [14, 63, 66]. In our study, two cases revealed no adenoma on EXP, however the tissue samples subsequently obtained from the IPSS suggesting side were consistent with pituitary adenoma on histologic examinations. In six cases, a cream-like substance was identified within the pituitary following incision, however histologic examination failed to demonstrate adrenocorticotropic adenoma in any of them. Nonetheless, 5 of the 6 patients went into remission following surgery, potentially due to the small size of tissue samples obtained which in turn made accurate histopathological assessment more difficult [14, 67].

In cases where EXP does not result in localization of an adenoma, surgical decision making becomes complicated. Generally, total hypophysectomy is not advisable due to high rates of endocrine complications as well as failing to provide significantly increased remission rates over partial hypophysectomy [62, 68]. In this scenario, multiple studies have recommended partial hypophysectomy based on IPSS lateralization as the next best step in management [63, 69]. Carr et al. suggested the advantage of 2/3 gland resection on remission rate in MRI-negative CD [60], but as previously discussed, IPSS may incorrectly lateralize adenomas, and thus surgeons should be hesitant when making decisions regarding tumor lateralization based solely on BIPSS data [17, 49]. Moreover, both adenomectomy and hypophysectomy are not without risks and potential complications. Surgical aggressiveness is correlated with increased likelihood of pituitary loss-of-function, supported by literature showing that the larger the amount of resection, the higher the rate of hypopituitarism. It has been reported that patients undergoing adenomectomy, hemi-hypophysectomy, and-total hypophysectomy had mean rates of hypopituitarism of 6.6, 20.2, and 80.2%, respectively [63, 70, 71]. As most CD patients are females of reproductive age, preserving child-bearing capacity is an important consideration, one which results in reluctance to perform hemi-hypophysectomy. In our series, we performed selective adenectomy when distinct adenomas were found, and in the cases where no adenoma was detected, meticulous EXP of pituitary gland bilaterally was performed. Subsequently, if EXP was inconclusive, a vertical median incision was made near the pituitary stalk to explore central part of the gland, which is believed to be the nest for adrenocorticotropic cells. Although an important step in localizing the adenoma, this also likely explains the high rate of postoperative DI in our study. No additional hemi-hypophysectomy was performed during the initial surgery in our study. With this technique, we achieved acceptable results with regards to remission rates, and none of our patients experienced panhypopituitarism in postoperative follow-ups. In one patient where CD recurred 2 years post-operatively, inadequate bony exposure and limited visualization of the medial wall of the right cavernous sinus resulted in failure to identify the adenoma during the initial surgery, further supporting the strategy of creating extensive exposure of the operative field in MRI-negative CD. Another possible reason for recurrence in this patient would be growth of a previously undetected microadenoma.

## Conclusion

Surgical treatment of MRI-negative Cushing’s disease is a demanding scenario necessitating multidisciplinary management. An experienced neurosurgeon working in collaboration with an endocrinologist should specify the most likely region of the tumor via IPSS. Additionally, surgical exploration of the pituitary is an invaluable tool in identifying adenomas while reducing the need for aggressive hypophysectomy, thus decreasing the likelihood of complications. Although MRI-negative Cushing’s disease presents significant challenges to neurosurgeons, surgical management remains essential in achieving remission.

## Data Availability

The authors confirm that the data supporting the findings of this study are available within the article.
